# Prepatellar Bursal Infection Caused by *Mycobacterium tuberculosis* with an In Situ Total Knee Arthroplasty: A Case Report and Comprehensive Literature Review

**DOI:** 10.1155/2019/4536714

**Published:** 2019-01-02

**Authors:** Abdulrahman D. Algarni

**Affiliations:** Department of Orthopedic Surgery, King Saud University, Riyadh, Saudi Arabia

## Abstract

Prepatellar bursal infection is a rare occurrence. The incidence of tuberculosis, including musculoskeletal type, is increasing. We present a case of isolated prepatellar bursal swelling associated with a discharging sinus; the condition developed in an elderly patient 4 years after total knee arthroplasty. Aspiration of the bursa revealed acid-fast bacilli on Ziehl–Neelsen staining, typical of *Mycobacterium tuberculosis*; this was confirmed later on culture. The patient was successfully treated with a 6-month course of antituberculous chemotherapy. To the best of our knowledge, only two previous cases of tuberculous prepatellar bursal infection have been reported in English literature. Our case illustrates the importance of considering tuberculous prepatellar bursal infection in the differential diagnosis of anterior knee swelling. All physicians treating patients with musculoskeletal disease should be aware of the possibility of this diagnosis and maintain a high index of suspicion; this is especially true in areas where tuberculosis is still endemic and in high-risk patients, such as the elderly.

## 1. Introduction

Prepatellar bursitis is common among people involved in occupations that require prolonged kneeling or crawling. Infection of the prepatellar bursa, however, is a rare occurrence. The risk factors for infection include a history of repetitive skin trauma, such as during prolonged kneeling, repeated local steroid injections, immunocompromised status, and previous instances of bursitis [1]. Unlike hematogenous seeding in septic arthritis, local trauma and the direct transcutaneous route is the proposed mechanism of infection for infected bursitis, probably because of the poor blood supply to the bursa [2]. Approximately, 80% of the cases of infected prepatellar bursitis are caused by *Staphylococcus aureus* and 15% by streptococcal species [2]. Rarely, atypical mycobacteria, *Brucella*, and fungi have been isolated as the causative organisms [3–6].

Existing literature suggests that the incidence of tuberculosis is increasing, both in developing and developed countries [7]. This increase is attributed to the rise in the number of immunocompromised patients, including the elderly and those with acquired immunodeficiency syndrome. Musculoskeletal involvement occurs in approximately 1%–5.2% of cases. Bursal tuberculous infection occurs in a small percentage of these cases and is mostly reported to occur in the greater trochanter bursa [8].

In this report, we describe a rare case of isolated prepatellar bursal infection with typical *Mycobacterium tuberculosis* and present a review of previously published cases in the English literature. The patient gave an informed consent that anonymous data and pictures concerning his case would be submitted for publication.

## 2. Case Presentation

A 74-year-old male patient underwent left total knee arthroplasty (TKA) at our institution for degenerative knee osteoarthrosis. He had an uneventful postoperative course with no history of delayed wound healing or persistent drainage. Four years later, he presented with a 2-month history of a gradually developing painless swelling over the anterior aspect of the operated knee; the swelling was associated with a small sinus that was extruding a straw-coloured fluid. He had no history of fever, decreased appetite, or weight loss. He had no other musculoskeletal, respiratory, or systemic symptoms of note. He had no history of antecedent trauma, recent travel, or contact with infectious diseases. The patient is a known hypertensive, but the blood pressure was well controlled with treatment, and he is otherwise healthy. He is a retired teacher with no history of involvement in activities requiring excessive kneeling. He is ambulatory in his community and can walk comfortably with the assistance of a cane.

**Figure 1:**
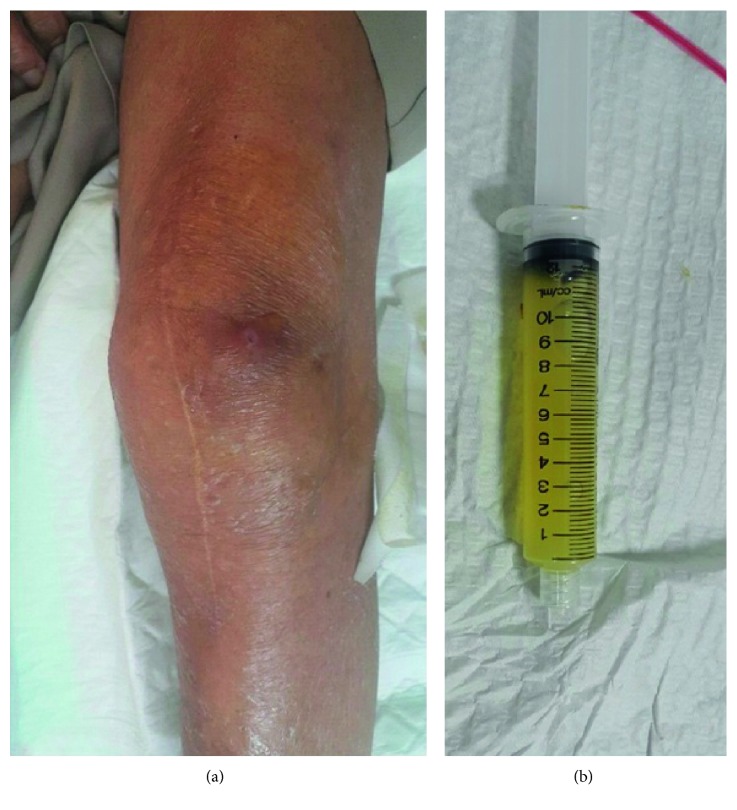
(a) Clinical photograph of the left knee showing the prepatellar swelling and the discharging sinus. (b) A syringe showing the colour of the aspirated fluid.

The patient's general physical examination results were within normal limits; positive physical findings were limited to the involved knee. There was an anterior knee swelling involving mainly the prepatellar area, approximately 7 cm in diameter, fluctuant, and not tender to palpation, with minimal surrounding erythema; the erythema was present mainly at the punctum. The punctum was draining a yellowish discharge on pressure (Figure 1(a)). There was no bony tenderness at the patella, distal femur, or proximal tibia. There was no detectable knee effusion, instability, or crepitus. The range of motion was well preserved (5–110°), as it was a prosthetic knee. It was only painful at the end of flexion as this movement compressed the prepatellar bursa.

**Figure 2:**
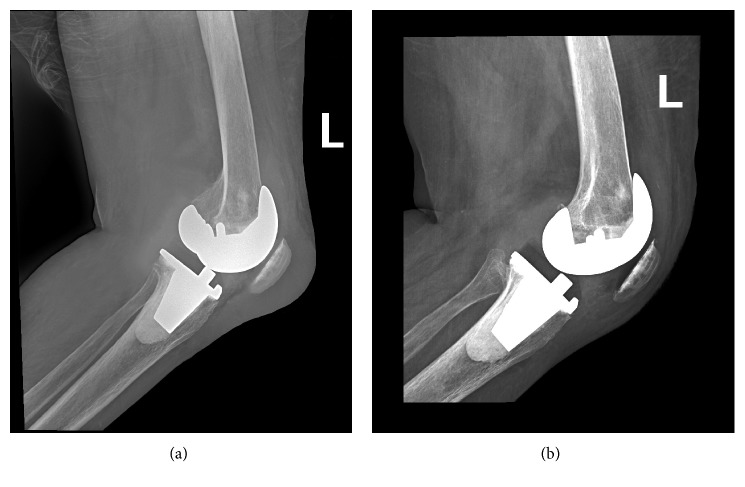
(a) Plain lateral view radiograph of the left knee showing the prepatellar soft tissue swelling and otherwise well-fixed prosthesis with no signs of osteolysis at the bone-prosthesis interface or at the patella. (b) Plain lateral view radiograph of the same knee at the 6-year follow-up showing regression of the swelling and otherwise normal appearance of the prosthetic knee and the patella.

Plain radiographs of the knee showed a prepatellar soft tissue swelling (Figure 2(a)). There were no obvious bony changes, osteolysis, or loosening at the bone-prosthesis interface. Needle aspiration of the prepatellar bursa yielded 50 mL of slightly turbid straw-coloured yellowish fluid that was sent as per our protocol for cell count determination, microcrystal analysis, Gram staining, and Ziehl–Neelsen (ZN) staining for acid-fast bacilli (Figure 1(b)). Cultures for aerobic and anaerobic bacteria, *Mycobacterium tuberculosis*, *Brucella*, and fungi were requested.

Considering the patient's age, chronic presentation, and the presence of sinus, he was admitted with a diagnosis of infected prepatellar bursitis. The primary aims of admission were wound dressing, awaiting aspiration results, and further work-up. Aspiration results revealed normal cell counts, no crystals, and no organisms on Gram stain. To our surprise, ZN stain revealed acid-fast bacilli consistent with typical tuberculous infection; the bacterium was confirmed on culture 6 weeks later as *Mycobacterium tuberculosis*-sensitive to rifampicin, isoniazid, ethambutol, and streptomycin. Cultures for bacteria, *Brucella*, and fungi were negative. Aspiration was repeated and yielded similar results. The blood work-up showed normal total and differential white blood cell count and slight elevation of both erythrocyte sedimentation rate (ESR) (80 mm/hr; normal: 30–70 mm/hr) and C-reactive protein (CRP) (8 mg/L; normal: 0–4 mg/L). Tuberculin skin test revealed a negative result (<5 mm induration at 72 hrs), and further work-up including chest radiograph and echocardiogram revealed no evidence of systemic disease. A triple three-phase bone scan displayed normal uptake both at the bone-prosthesis interface and at the patella. An infectious disease consult was obtained. The patient was started on rifampicin (600 mg/day), isoniazid (300 mg/day), pyrazinamide (1500 mg/day), and ethambutol (800 mg/day) for 2 months and continued on the same dosage of rifampicin and isoniazid to complete a 6-month course.

Three weeks later, the swelling significantly subsided in size and the sinus healed; therefore, the patient was discharged. He was reviewed at 6-week intervals at both the orthopaedic and infectious disease clinics for clinical progression and for any side effects of the medication. At the most recent 6-year follow-up, he was doing well with no evidence of local recurrence or prosthetic loosening (Figure 2(b)).

## 3. Discussion

The subcutaneous prepatellar bursa is a synovial bursa that lies between the skin and the patella. It is anatomically distinct from the superficial infrapatellar bursa, which lies in front of the patellar tendon and tibial tubercle. Sometimes, the two bursae are collectively referred to as the prepatellar bursa, which is inaccurate [1]. Standard textbooks of human anatomy describe the prepatellar bursa as a unilaminar or bilaminar bursa; however, Dye et al. found in a cadaveric study that the bursa has a trilaminar structure in 93% of knees [9, 10]. The three layers are a superficial subcutaneous layer, which lies between the subcutaneous tissue and an extension of the fascia lata, called the transverse superficial fascia; an intermediate subfascial layer, localized between the transverse superficial fascia and an oblique fascia formed by fascial extension of the vastus lateralis and medialis muscles; and a deep subaponeurotic layer, situated between the intermediate oblique fascia and the deep longitudinal fibres of the rectus femoris tendon [10].

Mycobacterial infection of the prepatellar bursa has been rarely reported. To date, only five cases have been reported in the English literature [4–6, 11, 12]. Of these 5, only 2 showed typical *Mycobacterium tuberculosis* infection, the rest showed atypical mycobacteria. Stewart reported the first case of mycobacterial tuberculous infection of the prepatellar bursa [11]. Schickendantz and Watson described another case of infected prepatellar bursa secondary to *Mycobacterium tuberculosis* infection [12]. Other authors published three similar cases due to infection with atypical mycobacteria; two of them were due to *Mycobacterium marinum (balnei)* and one due to *Mycobacterium fortuitum* [4–6]. In this report, we presented a third case of isolated mycobacterial tuberculous infection of the prepatellar bursa in an elderly patient with an in situ TKA.

The diagnosis of tuberculous prepatellar bursal infection is often difficult and requires a high index of clinical suspicion. Along with clinical assessment for possible intraarticular and/or patellar involvement, sufficient musculoskeletal imaging work-up is important. Owing to the presence of prosthesis and unavailability of prosthesis-compatible magnetic resonance imaging (metal artefact reduction sequence- (MARS-) MRI) at our institution, we were unable to perform an MRI, which would have enabled better delineation of the soft tissues and detect any possible early bony changes. MacLean and Kulkarni reported a case of tuberculous infection of the patella that presented as prepatellar bursitis [13]. There was no evidence of patellar or prosthetic joint involvement in our patient. Aspiration is helpful and is usually required for diagnosis. Unless special staining or polymerase chain reaction (PCR) test reveals the organism, culture requires 6 weeks of incubation to grow; this can further delay the diagnosis and treatment. It was fortunate in the present patient that ZN staining revealed the organism, as PCR testing is not available in most institutions, including ours. If a biopsy tissue is obtained, histopathological examination is nonspecific and demonstrates evidence of chronic inflammation and caseating granulomas [8].

Empiric antituberculous chemotherapy is recommended if the clinical scenario and the work-up are suggestive of this diagnosis, regardless of the diagnostic team's ability to isolate the organism [14]. A general work-up for systemic tuberculous disease is also mandated. In tuberculous infection, as in this case, ESR and CRP are often only mildly elevated or can even be normal. Our patient had no evidence of systemic tuberculosis nor did he have a previous known contact with the disease despite being from a country where tuberculosis is still prevalent. We hypothesised that transcutaneous inoculation of mycobacteria was the probable mechanism for bursal infection, similar to what was reported in previously mentioned cases [4–6, 11, 12].

Multidrug antituberculous chemotherapy is considered the mainstay of treatment; surgical drainage and debridement are performed in case of a pointing abscess [14]. A well-placed incision is recommended; generally, the lateral parapatellar incision is used. Schickendantz and Watson performed a total bursectomy in their Case [12]. Quayle and Robinson proposed a technique in which only the posterior wall is resected, leaving the anterior wall adherent to the subcutaneous tissue; they recommended this to reduce the skin-related complications of total bursectomy, [15]. Our patient was effectively treated only with antituberculous chemotherapy.

## 4. Conclusion

Although tuberculous prepatellar bursal infection is a rarely reported entity, this case illustrates that it should be considered in the differential diagnosis of anterior knee swelling that does not respond to conventional management. Physicians who treat patients with musculoskeletal disease should be aware of the possibility of this diagnosis and maintain a high index of suspicion, especially in areas where tuberculosis is still endemic as well as in high-risk groups such as elderly patients.

## References

[1] Aaron D. L., Patel A., Kayiaros S., Calfee R. (2011). Four common types of bursitis: diagnosis and management. *American Academy of Orthopaedic Surgeon*.

[2] Zimmermann B. W., Mikolich D. J., Ho G. (1995). Septic bursitis. *Seminars in Arthritis and Rheumatism*.

[3] Kelley P. J., Weed L. A., Lipscomb P. R. (1963). Infection of tendon sheaths, bursae, joints, and soft tissues by acid-fast bacilli other than tubercle bacilli. *Journal of Bone and Joint Surgery*.

[4] Winter F. E., Runyon E. H. (1965). Prepatellar bursitis caused by mycobacterium marinum (balnei). *Journal of Bone and Joint Surgery*.

[5] Kelly P. J., Karlson A. G., Weed L. A., Lipscomb P. R. (1967). Infection of synovial tissues by mycobacteria other than mycobacterium tuberculosis. *Journal of Bone and Joint Surgery*.

[6] Wallach J. C., Delpino M. V., Scian R., Deodato B., Fossati C. A., Baldi P. C. (2010). Prepatellar bursitis due to Brucella abortus: case report and analysis of the local immune response. *Journal of Medical Microbiology*.

[7] Jitmuang A., Yuenyongviwat V., Charoencholvanich K., Chayakulkeeree M. (2017). Rapidly-growing mycobacterial infection: a recognized cause of early-onset prosthetic joint infection. *BMC Infect Dis*.

[8] Crespo M., Pigrau C., Flores X. (2009). Tuberculous trochanteric bursitis: report of 5 cases and literature review. *Scandinavian Journal of Infectious Diseases*.

[9] Netter F. H., Colacino S. (1989). *Atlas of Human Anatomy*.

[10] Dye S. F., Campagna-pinto D., Dye C. C., Shifflett S., Eiman T. (2003). Soft-tissue anatomy anterior to the human patella. *Journal of Bone and Joint Surgery-American Volume*.

[11] Stewart W. J. (1933). Tuberculous bursitis without adjacent joint involvement following trauma. *Journal of Bone and Joint Surgery*.

[12] Schickendantz M. S., Watson J. T. (1990). Mycobacterial prepatellar bursitis. *Clinical Orthopaedics and Related Research*.

[13] MacLean S., Kulkarni S. (2013). Tuberculosis of the patella masquerading as prepatellar bursitis. *Annals of The Royal College of Surgeons of England*.

[14] Hogan J. I., Hurtado R. M., Nelson S. B. (2017). Mycobacterial musculoskeletal infections. *Infectious Disease Clinics of North America*.

[15] Quayle J. B., Robinson M. P. (1978). An operation for chronic prepatellar bursitis. *Journal of Bone and Joint Surgery*.

